# Thyroid Imaging Reporting and Data System Score Combined with the New Italian Classification for Thyroid Cytology Improves the Clinical Management of Indeterminate Nodules

**DOI:** 10.1155/2017/9692304

**Published:** 2017-03-01

**Authors:** Salvatore Ulisse, Daniela Bosco, Francesco Nardi, Angela Nesca, Eleonora D'Armiento, Valeria Guglielmino, Corrado De Vito, Salvatore Sorrenti, Daniele Pironi, Francesco Tartaglia, Stefano Arcieri, Antonio Catania, Massimo Monti, Angelo Filippini, Valeria Ascoli

**Affiliations:** ^1^Department of Surgical Sciences, “Sapienza” University of Rome, 00161 Rome, Italy; ^2^Department of Radiological, Oncological and Anatomo-Pathological Sciences, “Sapienza” University of Rome, 00161 Rome, Italy; ^3^Department of Experimental Medicine, “Sapienza” University of Rome, 00161 Rome, Italy; ^4^Department of Public Health and Infectious Disease, “Sapienza” University of Rome, 00161 Rome, Italy

## Abstract

The new Italian cytological classification (2014) of thyroid nodules replaced the TIR3 category of the old classification (2007) with two subclasses, TIR3A and TIR3B, with the aim of reducing the rate of surgery for benign diseases. Moreover, thyroid imaging reporting and data system (TI-RADS) score appears to ameliorate the stratification of the malignancy risk. We evaluated whether the new Italian classification has improved diagnostic accuracy and whether its association with TI-RADS score could improve malignancy prediction. We retrospectively analyzed 70 nodules from 70 patients classified as TIR3 according to the old Italian classification who underwent surgery for histological diagnosis. Of these, 51 were available for cytological revision according to the new Italian cytological classification. Risk of malignancy was determined for TIR3A and TIR3B, TI-RADS score, and their combination. A different rate of malignancy (*p* = 0.0286) between TIR3A (13.04%) and TIR3B (44.44%) was observed. Also TI-RADS score is significantly (*p* = 0.003) associated with malignancy. By combining cytology and TI-RADS score, patients could be divided into three groups with low (8.3%), intermediate (21.4%), and high (80%) risk of malignancy. In conclusion, the new Italian cytological classification has an improved diagnostic accuracy. Interestingly, the combination of cytology and TI-RADS score offers a better stratification of the malignancy risk.

## 1. Introduction

Palpable thyroid nodules are present in about 4–7% of the adult population in countries with adequate iodine intake and up to 20% in iodine insufficient areas [1, 2]. High-resolution ultrasound can detect thyroid nodules in 19–68% of randomly selected individuals with higher frequencies in women and elderly [2, 3]. Since about 5% of patients affected by thyroid nodules harbour a malignant lesion, the first aim in their evaluation is to exclude malignancy [4]. To this end, fine-needle aspiration cytology (FNAC) represents the diagnostic cornerstone because of its diagnostic accuracy, reproducibility, and cost-effectiveness [5–8]. However, FNAC is characterized by a grey diagnostic area in which the indeterminate cytology precludes a distinction between benign and malignant lesions [9]. Surgical excision, frequently necessary to obtain a definitive diagnosis, shows that about 80% of these patients harbour a benign lesion [9]. In order to reduce unnecessary thyroidectomy, a number of instrumental and molecular diagnostic approaches have been proposed [10–27]. In addition, new classification systems for thyroid cytology have been designed. In particular, in the Bethesda System for Reporting Thyroid Cytopathology (BSRTC), indeterminate nodules have been subcategorized in atypia or follicular lesions of undetermined significance (AUS/FLUS) and in follicular neoplasms or suspicious for follicular neoplasms (FN/SFN) which are expected to correspond to different rate of malignancy with different clinical action required [23]. In 2009, the British Thyroid Association-Royal College of Pathologists (BTA-RCPath) revised the previous British reporting system already in use in UK since 2007 along the lines of the BSRTC and split the Thy 3 category in Thy 3a and Thy 3f [28]. In 2014, the Italian Society for Anatomic Pathology and Cytology (SIAPEC) together with the Italian Thyroid Association (AIT) modified the previous thyroid cytology classification of 2007, by replacing the TIR3 class with two new subclasses, TIR3A and TIR3B [24–26]. The latter are comparable both to the BSRTC AUS/FLUS and FN/SFN classes and to the BTA-RCPath classes Thy 3a and Thy 3f [26]. However, unlike the BSRTC and BTA-RCPath, the SIAPEC extends TIR3B to include those cases with “mild/focal nuclear atypia” suggestive of papillary carcinoma that are expected to be at higher risk of malignancy [24].

The present study was conducted with the aim of evaluating whether the malignancy rate of TIR3A and TIR3B differs, thus improving the clinical management of patients with lesions classified in the TIR3 category in the previous classification. To this end, cytological smears of 51 nodules from patients thyroidectomized following TIR3 diagnosis were independently reevaluated by the same three cytopathologists, who made the initial TIR3 cytological diagnosis, according to the new SIAPEC 2014 classification. In addition, we evaluated whether the thyroid imaging reporting and data system (TI-RADS) score, either alone or in combination with the SIAPEC 2014 cytological diagnosis, could be of any value in predicting malignancy.

## 2. Materials and Methods

### 2.1. Patients

The series comprises 70 patients (52 females and 18 males, median age 58, range 13–77 yr) who underwent fine-needle aspiration cytology (FNAC) in the period between January 2005 and December 2013. Patients provided their written informed consent. All of them had a TIR3 diagnosis based on the old Italian cytological classification (SIAPEC 2007) and were submitted to surgical excision for histological diagnosis, as shown in Table 1. Of the 70 cases, cytological smears were available for 51 patients. Of the latter, one patient with histological diagnosis of uncertain malignant potential was excluded from the analysis. All smears were independently reevaluated by the same three cytopathologists (FN, VA, and DB), who made the initial TIR3 cytological diagnosis, according to the new SIAPEC 2014 classification, blinded for histology.

### 2.2. Ultrasound and Color-Flow Doppler

Thyroid ultrasonography (US) was performed in 69 patients using a Toshiba Aplio XV system equipped with a linear transducer (PLT-805AT). The nodule classification was based on echogenicity and echostructure: solid hypoechoic, solid isoechoic, solid hyperechoic, mixed, or anechoic. Anteroposterior (APD), transverse (TD), and longitudinal (LD) diameters of the nodules were used to obtain the volume of the nodules, based on the formula of ellipsoid: Volume = APD × *TD* × *LD* × *π*/6.

Nodule margin was defined regular or irregular. Microcalcifications, defined as hyperechoic spots < 22 *mm*, were recorded. The pattern of nodular vascular signal was evaluated by color-flow Doppler (CFD) and defined as CFD 1, as an absent signal; CFD 2, as a perinodular spot signal; and CFD 3, as a perinodular and/or intranodular signal. Ultrasound examinations were performed by two observers (AN, EDA) with an agreement on the US and CFD characteristics greater than 95%. US features, such as hypoechogenicity, irregular margins, microcalcifications, and taller-than-wide shape, were used to calculate the TI-RADS score as described by Kwak et al. [16].

### 2.3. Fine-Needle Aspiration Cytology

All patients were instructed not to take aspirin or any other anticoagulants 5 days prior to thyroid nodule aspiration. A 23–27 gauge needle, attached to 20 ml plastic syringes, was used to aspirate nodules. All aspirates were smeared directly on 4–6 glass slides and stained by May-Grunwald-Giemsa and Papanicolaou. Cytological specimens of the 70 patients had been evaluated by three cytopathologists (FN, VA, and DB) from the same institution. All 70 nodules had a TIR3 cytological diagnosis, based on the SIAPEC 2007 classification: TIR1, nondiagnostic; TIR2, negative for malignant cells; TIR3, indeterminate (follicular lesion); TIR4, suspicious for malignancy; and TIR5, diagnostic of malignancy [24]. Of these 70 cases, cytological smears were available for 51 nodules, which were reevaluated collegially by the same three cytopathologists according to the new SIAPEC 2014 classification [24]. In particular, these cases were reevaluated and classified either as TIR3A (low-risk indeterminate lesion) or as TIR3B (high-risk indeterminate lesion). Discordant diagnosis was resolved by a consensus review.

### 2.4. Histological Outcome

Histological diagnoses of patients who had undergone surgery were used as gold standard for correlation with the cytological interpretations according to the WHO classification currently in use (Table 1) [29].

### 2.5. Statistical Analysis

Patients' age and mean nodular volume in benign versus malignant thyroid nodules were compared by the nonparametric Mann–Whitney *U* test, while clinical, US, and CFD characteristics were compared by the *χ*^2^ test or the Fisher exact test. The statistical significance was set at *p* < 0.05.

## 3. Results

Following total thyroidectomy, histological diagnosis showed that, of the 70 patients with indeterminate lesions, 17 (24.3%, 13 females and 4 males) had a malignant lesion. Of these, 2 had follicular thyroid carcinoma (FTC) and 15 papillary thyroid carcinoma (PTC), of which 12 classical and 3 follicular variants, and 1 had a well-differentiated tumor of uncertain malignant potential. The remaining 52 patients (74.3%, 38 females and 14 males) were affected by benign lesions, including 10 follicular adenoma, 40 nodular hyperplasia, 1 Hürthle adenoma, and 1 chronic lymphocytic thyroiditis.

We initially evaluated the role of gender, patient's age at diagnosis, size of the lesion, ultrasound, and color-flow Doppler features in predicting malignancy. No significant association was observed for gender, median age at diagnosis, echostructure, nodularity, and color-flow Doppler between benign and malignant lesions (Table 2). On the other hand, lower median nodule's volume (*p* = 0.016), taller-than-wide nodule's shape (*p* = 0.046), irregular margin (*p* = 0.008), microcalcifications (*p* = 0.043), and hypoechogenicity (*p* = 0.021) are associated with nodule malignancy (Table 2).

We next evaluated whether the TI-RADS score is associated with lesion's malignancy and found a positive correlation between TI-RADS score and risk of malignancy (*p* = 0.003) (Table 3).

Since cytological smears were available for 51 of the 70 patients, they were independently reevaluated by the same three cytopathologists who provided the initial diagnosis according to the new SIAPEC 2014 classification, in which the TIR3 category was replaced by two subclasses, TIR3A and TIR3B. A concordant diagnosis was reached in 32 cases. The 19 (37.2%) cases with discordant diagnosis were resolved by a consensus review. From the 51 cases, however, the single patient with histological diagnosis of uncertain malignant potential was excluded from the analysis. The results showed a difference in the rate of malignancy (*p* = 0.0286) between the TIR3A and TIR3B lesions (Table 4).

In the attempt to stratify indeterminate lesions according to the risk of malignancy, we combined cytology and TI-RADS score. In particular, the TIR3A and TIR3B categories were dichotomized based on TI-RADS score, as reported in Table 5. As it may be noticed, the TIR3A combined with the TI-RADS scores 3, 4a, and 4b showed a low malignancy rate (8.3%). On the other hand, the TIR3A combined with the TI-RADS scores 4c and 5, as well as the TIR3B combined with the TI-RADS scores 3, 4a, and 4b, showed an intermediate risk of malignancy (20.9%). Lastly, the TIR3B combined with the TI-RADS scores 4c and 5 showed a high risk of malignancy (80%). These results lead us to propose a new stratification of the risk of malignancy for indeterminate lesions in low, intermediate, and high as reported in Table 6.

## 4. Discussion

Over the last years, major efforts have been made to generate new classification systems for thyroid cytology in order to ameliorate the diagnostic stratification of nodules with indeterminate cytology, a grey area of fine-needle aspiration cytology [23–28]. In particular, all the new systems have subcategorized the indeterminate lesions into two subclasses which are expected to correspond to different rate of malignancy. In particular, in the AUS/FLUS category of the BSRTC, in the Thy 3a category of the BTA-RCPath, and in the category TIR3A of the SIAPEC 2014, a low rate of malignancy (5–15%) is expected, while a significantly higher rate, comprised between 15% and 30%, is expected in the FN/SFN category of the BSRTC, in the Thy 3f category of the BTA-RCPath, and in the TIR3B category of the SIAPEC 2014. Importantly, these new diagnostic classifications are expected to drive the clinical management of patients. In fact, in view of the relative low rate of malignancy of the AUS/FLUS, Thy 3a and TIR3A categories, follow-up of the patients and FNAC repetition is suggested. On the other hand, the relative high rate of malignancy observed in the FN/SFN, Thy 3f, and TIR3B recommends surgery as the preferential option [23–28]. However, a study from Brophy and colleagues, using the BTA-RCPath classification system on 151 Thy 3 nodules with histological diagnosis, found no difference in the malignancy rate of lesions classified as Thy 3a and Thy 3f [30]. In particular, although the authors observed a slightly higher malignancy rate in Thy 3f cases (17.9%) than in Thy 3a cases (13.4%), this difference was not significant. In addition, similar findings emerged from a recent meta-analysis of 51 studies, using the BSRTC classification system on a total of 4475 AUS/FLUS and 3202 FN/SFN nodules, showing a 27% rate of malignancy for the AUF/FLUS FNAC and 31% for the FN/SFN FNACs [31]. In the latter of relevance is the high rate of malignancy observed in the AUS/FLUS categories, with respect to the expectations of BSRTC. In this context, studies attempting to perform a 2-tier subclassification of the AUS/FLUS categories in order to achieve a more accurate estimate of the risk of malignancy have been reported [32, 33].

The data reported here are not in agreement with the above studies because in our series, a significant difference could be observed between the rates of malignancies of TIR3A and TIR3B cases. In fact, in TIR3A category, we found a slight higher rate of malignancy (13%), with respect to the expected one (<10%), while TIR3B category had a higher malignant rate (44%), with respect to the expected one (20%) [26]. These data demonstrated that the new SIAPEC 2014 provides a better stratification of the malignancy risk for indeterminate lesions, with respect to previous SIAPEC 2007 classification [26]. With respect to the BSRTC and BTA-RCPath, it must be emphasized that, in the SIAPEC 2014 Italian classification, the TIR3B subcategory includes samples characterized by nuclear alterations suggestive of papillary carcinoma that are too mild or focal to be included in the TIR4 category. Even if our data derive from a relatively small series of cases, they are in line with the referred increasing evidence that the AUS/FLUS with cytological atypia is the AUS/FLUS subcategory most frequently associated with malignancy [34]. It has to be said, however, that the consensus review of the cytological smears by three different cytopathologists (also suggested by the BSRTC in challenging cases) could have affected the outcome of TIR3 subclassification in TIR3A and TIR3B, representing a possible confounding factor with respect to one observer performance [35, 36]. In this context, it is also worth mentioning that, in 37.2% (in 19 out of 51) of cases, a discordant diagnosis was made by the three different cytopathologists, which was then resolved by a consensus review of the cytological smears. This is not surprising since it is well known in the literature that the major interobserver discordance is observed in the indeterminate lesions [37].

However, also with this classification, more than half of TIR3B patients undergo unnecessary surgery. The ability of TI-RADS score in predicting thyroid nodule malignancy has been demonstrated [17]. This was confirmed in the present study also for indeterminate lesions, in which a significant association between high-risk TI-RADS score and malignancy is observed. For this reason, we attempted to ameliorate the accuracy of the SIAPEC 2014 thyroid cytological classification by combining it with the TI-RADS score. In particular, dichotomizing the TIR3A and TIR3B categories based on low-risk TI-RADS score (3, 4a, and 4b) and high-risk TI-RADS score (4c and 5), the risk of malignancy for indeterminate lesions could be stratified in three classes: low (below 10%), intermediate (about 20%), and high (about 80%). Similar results were recently reported by Maia and colleagues using the Bethesda system and by Chng and colleagues using the BTA-RCPath system [38, 39].

## 5. Conclusions

Compared to the old SIAPEC 2007, the new SIAPEC 2014 thyroid cytological classification has improved diagnostic accuracy, reducing the numbers of patients with indeterminate lesions requiring surgery. The combination of SIAPEC 2014 thyroid cytological classification and TI-RADS score could offer a better stratification of the malignancy risk suggesting a conservative approach for low-risk class and a surgical approach for high-risk class. For patients with intermediate risk, a careful evaluation of risk factors for thyroid malignancy and a close follow-up is recommended.

## Figures and Tables

**Table 1:** Demographic, ultrasonographic, cytological, and histological parameters of 70 patients affected by indeterminate thyroid lesions. TI-RADS, thyroid imaging reporting and data system; FTC, follicular thyroid cancer; FA, follicular adenoma; CV-PTC, classical variant of papillary thyroid carcinoma; FV-PTC, follicular variant of papillary thyroid carcinoma; NH, nodular hyperplasia; CLT, chronic lymphocytic thyroiditis; WDT-UMP, well-differentiated tumor-uncertain malignant potential; HA, Hürthle adenoma. Nodule volume is expressed in milliliters.

Number	Sex	Age	Nodule volume	SIAPEC 2007	SIAPEC 2014	Nodule histological diagnosis	TI-RADS score
1	M	20	2.268	TIR3	TIR3B	FTC	4c
2	F	24	0.252	TIR3	TIR3A	FA	4c
3	F	28	4.18	TIR3	TIR3A	CV-PTC	4c
4	F	35	0.432	TIR3	TIR3A	FV-PTC	4c
5	M	36	0.99	TIR3	TIR3A	NH	4c
6	F	41	0.495	TIR3	TIR3B	CV-PTC	5
7	F	42	1.607	TIR3	TIR3B	NH	4b
8	F	43	0.45	TIR3	TIR3B	CV-PTC	4c
9	F	50	0.243	TIR3	TIR3B	CV-PTC	4c
10	M	52	2.2	TIR3	TIR3A	NH	4b
11	M	54	1.296	TIR3	TIR3A	NH	4b
12	F	55	2.66	TIR3	TIR3B	NH	4b
13	F	55	4.9	TIR3	TIR3A	NH	4a
14	F	58	1.26	TIR3	TIR3A	FA	4c
15	M	58	4.568	TIR3	TIR3A	NH	3
16	F	61	0.18	TIR3	TIR3B	NH	4c
17	F	62	3.658	TIR3	TIR3A	NH	4c
18	F	64	0.99	TIR3	TIR3A	CLT	3
19	F	66	1.08	TIR3	TIR3A	NH	4c
20	F	68	0.096	TIR3	TIR3B	CV-PTC	4c
21	F	69	0.2	TIR3	TIR3B	NH	4b
22	F	69	0.792	TIR3	TIR3A	WDT-UMP	4c
23	F	72	1.08	TIR3	TIR3A	FA	4b
24	F	75	3.12	TIR3	TIR3B	CV-PTC	4b
25	F	52	0.123	TIR3	TIR3B	NH	4c
26	F	63	2.025	TIR3	TIR3B	NH	4a
27	F	64	0.264	TIR3	TIR3B	CV-PTC	4b
28	F	73	0.49	TIR3	TIR3A	NH	4c
29	M	57	0.756	TIR3	TIR3B	CV-PTC	4b
30	F	60	0.484	TIR3	TIR3B	NH	4b
31	M	40	1.344	TIR3	TIR3B	NH	4a
32	F	57	0.24	TIR3	TIR3A	NH	4b
33	M	32	1.08	TIR3	TIR3B	NH	4a
34	F	45	0.196	TIR3	TIR3B	FV-PTC	4c
35	F	13	0.576	TIR3	TIR3A	NH	4c
36	F	27	8.58	TIR3	TIR3A	NH	4a
37	F	46	0.168	TIR3	TIR3B	CV-PTC	4c
38	F	58	2.04	TIR3	TIR3B	FA	4b
39	M	56	3.105	TIR3	TIR3B	NH	4a
40	F	63	0.75	TIR3	TIR3A	NH	4a
41	F	67	4.774	TIR3	TIR3B	FA	4b
42	M	49	0.364	TIR3	TIR3B	FV-PTC	4b
43	F	56	0.216	TIR3	TIR3B	CV-PTC	4c
44	F	64	0.96	TIR3	TIR3A	NH	4a
45	M	64	1.53	TIR3	TIR3B	NH	4b
46	F	73	1.755	TIR3	TIR3B	NH	4a
47	F	42	1.19	TIR3	TIR3A	NH	4c
48	F	77	2.7	TIR3	TIR3B	NH	4b
49	F	68	7.038	TIR3	TIR3A	FTC	4a
50	M	65	1.615	TIR3	TIR3A	FA	4c
51	F	70	0.88	TIR3	TIR3A	HA	4b
52	M	40	10.93	TIR3	—	NH	4b
53	F	63	1.02	TIR3	—	NH	4a
54	F	31	12.18	TIR3	—	NH	3
55	F	50	1.836	TIR3	—	NH	3
56	F	63	2.432	TIR3	—	NH	4a
57	F	66	1.224	TIR3	—	NH	4c
58	M	16	0.32	TIR3	—	FA	4a
59	M	24	1.368	TIR3	—	FA	4c
60	M	69	0.825	TIR3	—	NH	4b
61	F	43	0.336	TIR3	—	NH	4b
62	F	71	0.833	TIR3	—	NH	4b
63	F	40	2.835	TIR3	—	NH	4a
64	M	59	0.216	TIR3	—	CV-PTC	4c
65	F	70	0.41	TIR3	—	NH	4b
66	M	65	0.462	TIR3	—	FA	4c
67	F	36	0.16	TIR3	—	NH	3
68	F	39	0.484	TIR3	—	NH	4a
69	F	56	0.672	TIR3	—	CV-PTC	4c
70	F	67	4.641	TIR3	—	FA	3

**Table 2:** Association of clinical and ultrasonographic (US) features with histology of 69 nodules with indeterminate cytological diagnosis. CFD, color-flow Doppler.

Clinical and US features	Benign (*n* = 52)	Malignant (*n* = 17)	*p*
*Gender*			
Male	14 (26.29%)	4 (23.52%)	0.527
Female	38 (73.1%)	13 (76.47%)	

*Age (yr)*			
Median age	58 (13–77)	50 (20–75)	0.347

*Morphology*			
Taller than wide	7 (13.46%)	6 (35.3%)	**0.046**
Round/oval shape	45 (86.53%)	11 (64.70%)	

Median nodule volume (ml)	1.2 (0.12–12)	0.44 (0.1–8.6)	**0.016**

*Margin*			
Irregular	15 (28.8%)	11 (64.70%)	**0.008**
Regular	37 (71.1%)	6 (35.3%)	

*Microcalcification*			
Yes	9 (17.30%)	7 (41.2%)	**0.043**
No	43 (82.7%)	10 (58.9%)	

*Echogenicity*			
Hypoechogen	25 (48.1%)	13 (76.47%)	**0.021**
Isoechogen	19 (36.5%)	1 (5.88%)	

*Echostructure*			
Mixed	8 (15.4%)	3 (17.64%)	0.545
Solid	44 (84.6%)	14 (82.35%)	

*Color-flow Doppler*			
CFD 1	11 (21.15%)	5 (29.41%)	0.755
CFD 2	6 (11.5%)	1 (5.88%)	
CFD 3	35 (67.30%)	11 (64.70%)	

*Nodularity*			
Uninodular	20 (38.5%)	5 (29.41%)	0.356
Multinodular	32 (61.53%)	12 (70.6%)	

**Table 3:** Malignancy rate according to TI-RADS score in 69 indeterminate lesions.

TI-RADS score	Number of cases	Benign (*n*, %)	Malignant (*n*, %)	*p*
3	3	3 (100%)	0 (0%)	**0.003**
4a	16	16 (100%)	0 (0%)	
4b	24	19 (79.2%)	5 (20.8%)	
4c	25	14 (56%)	11 (44%)	
5	1	0	1 (100%)	

**Table 4:** Malignancy rate of the SIAPEC 2014 classification of 50 indeterminate lesions with indeterminate cytological diagnosis.

	Benign (*n* = 35)	Malignant (*n* = 15)	*p*
TIR3A (*n* = 23)	20 (86.96%)	3 (13.04%)	
TIR3B (*n* = 27)	15 (55.56%)	12 (44.44%)	**0.0286**

**Table 5:** Malignancy rate of indeterminate lesions by combining TI-RADS score with SIAPEC 2014 classification.

	TI-RADS categories	Number of cases	Benign (*n*)	Malignant (*n*)	Malignancy rate (%)	*p*
TIR3A	3; 4a; 4b	12	11	1	8.3	0.466
	4c; 5	11	9	2	18.2	

TIR3B	3; 4a; 4b	17	13	4	23.5	**0.007**
	4c; 5	10	2	8	80	

**Table 6:** Proposed stratification risk of malignancy in indeterminate lesions by combining TI-RADS score with SIAPEC 2014 classification.

Malignancy risk	Number of cases	Benign (*n*, %)	Malignant (*n*, %)	*p*
Low	12	11 (91.7%)	**1 (8.3%)**	**0.001**
TIR3A with TI-RADS categories 3; 4a; 4b				
Intermediate	28	22 (78.6%)	**6 (21.4%)**	
TIR3A with TI-RADS categories 4c; 5				
TIR3B with TI-RADS categories 3; 4a; 4b				
High	10	2 (20%)	**8 (80%)**	
TIR3B with TI-RADS categories 4c; 5				
